# 

**Published:** 1885-06

**Authors:** 


					﻿Whole Number,
CCLXXVII.
JUNE, 1885.
Number i i,
Volume XXIV.
THE
BUFFALO
Madicakaiid Surgical Journal.
EDITORS :
THOM. LOTHROP, M. ».
A. R. DAVIDSON, M. D.
Published by the Medical Journal Association.
No, B WEST CHIPPEWA ST.
Entered at the Post-Office at Buffalo, N. Y., as Second-class Mail Matter.
				

